# Surgical Planning after Neoadjuvant Treatment in Breast Cancer: A Multimodality Imaging-Based Approach Focused on MRI

**DOI:** 10.3390/cancers15051439

**Published:** 2023-02-24

**Authors:** Marco Conti, Francesca Morciano, Enida Bufi, Anna D’Angelo, Camilla Panico, Valerio Di Paola, Elisabetta Gori, Gianluca Russo, Giovanni Cimino, Simone Palma, Paolo Belli, Riccardo Manfredi

**Affiliations:** 1 Department of Bioimaging, Radiation Oncology and Hematology, UOC of Radiologia, Fondazione Policlinico Universitario A. Gemelli IRCSS, Largo A. Gemelli 8, 00168 Rome, Italy; 2 Institute of Radiology, Catholic University of the Sacred Heart, Largo A. Gemelli 8, 00168 Rome, Italy; 3 Radiology Unit, S. Maria Della Scaletta Hospital, Via Montericco 4, 40026 Imola, Italy

**Keywords:** neoadjuvant chemotherapy (NACT), locally advanced breast cancer (LABC), magnetic resonance imaging (MRI), breast-conservative surgery (BCS), conservative mastectomy with reconstruction (CMR), oncoplastic surgery (OPS), sentinel lymph node biopsy technique (SLNB), axillary lymphadenectomy (AL), selective axillary dissection (SAD), clipped lymph node (CL)

## Abstract

**Simple Summary:**

The effectiveness of neoadjuvant treatment has led to an expansion of its indications from locally advanced to highly chemo-sensitive early-stage breast cancers, aiming to increase conservative treatments, in place of more invasive surgery, and to improve long term outcomes. At the same time, the continuous development of diagnostic techniques necessitates continuous updating, due to their importance in tumoral staging and in the prediction of the response to treatment, as well as in surgical planning. With our review, we sought to discuss the strengths of the various imaging modalities; in particular, the role of magnetic resonance imaging, which is still the center of scientific debate in this setting. Moreover, we analyzed the evolution of surgical approaches to breast cancer in patients treated with neoadjuvant chemotherapy.

**Abstract:**

Neoadjuvant chemotherapy (NACT) today represents a cornerstone in the treatment of locally advanced breast cancer and highly chemo-sensitive tumors at early stages, increasing the possibilities of performing more conservative treatments and improving long term outcomes. Imaging has a fundamental role in the staging and prediction of the response to NACT, thus aiding surgical planning and avoiding overtreatment. In this review, we first examine and compare the role of conventional and advanced imaging techniques in preoperative T Staging after NACT and in the evaluation of lymph node involvement. In the second part, we analyze the different surgical approaches, discussing the role of axillary surgery, as well as the possibility of non-operative management after-NACT, which has been the subject of recent trials. Finally, we focus on emerging techniques that will change the diagnostic assessment of breast cancer in the near future.

## 1. Introduction

Neoadjuvant chemotherapy (NACT) today represents a cornerstone in the treatment of locally advanced breast cancer (LABC) and highly chemo-sensitive tumors, such as the triple-negative (TN) and HER2-positive subtypes, even at early stages [1,2,3,4,5]. Indications for NACT include clinical parameters (i.e., tumor size and phenotype), lymph node involvement, and high-grade disease. NACT has a key role in reducing tumor size, increasing the possibility of performing breast-conservative surgery (BCS) over conservative mastectomy with reconstruction (CMR) [6,7], and so reducing the need for axillary lymph node dissection [2,8,9]. Another benefit of NACT is evaluating the in vivo response, to consequently allow a tailored adjuvant chemotherapy [2,10,11].

The challenge of BCS is to obtain clear margins, in order to decrease the incidence of loco-regional recurrences and, at the same time, to preserve as far as possible the healthy tissue, for the best aesthetic result [1]. Currently no specific guidelines support the choice of the best type of surgery for post-NACT patients. Some authors have tried to define which factors can influence the therapeutic choice in the neoadjuvant setting, with multifocality disease, extensive microcalcifications, and a lobular histotype predictive for mastectomy [12,13,14,15].

Even on the axillary side, surgery is now able to propose more conservative treatments instead of lymphadenectomy, even in patients node-positive at diagnosis [16,17,18].

There is, furthermore, a great variability in the response of breast cancer to NACT: an accurate evaluation of the residual disease is crucial to ensure the best assessment of patients, reducing morbidity and the necessity for further surgical procedures. Nonetheless, the detection of a pathologically complete response (pCR), strictly linked to prognosis, could help clinicians to orient towards tailored treatments [19,20,21,22,23].

For these reasons, imaging techniques have a crucial role in surgical planning in post-NACT patients, in order to predict pathological response and disease extension. 

Physical examination, mammography (MX), ultrasound (US), and magnetic resonance imaging (MRI) are different techniques used to evaluate the residual tumor burden. The diagnostic accuracy of each of these is based on the ability to discriminate cancerous cells from the fibrosis resulting from biopsy procedures and chemotherapy, as well as from necrosis and fragmentation [24,25].

## 2. Role of Conventional Techniques in Preoperative T Staging after NACT

### 2.1. Mammography

The two principal features of mammography linked to a tumoral response after neoadjuvant therapies are changes in mass dimension and density, and the disappearance of microcalcifications. Unfortunately, these signs do not have a high accuracy in predicting pCR, making this technique unsuitable for preoperative staging after treatment. Some retrospective studies proved that mammography measurement had little consistency compared to pathological results in patients following NACT, with a mean concordance correlation coefficient (CCC) of only 0.55 [26,27].

In a prospective study published in 2020, the diagnostic accuracy of MX in predicting pCR post treatment was reported as having a sensitivity of 0.65, specificity of 0.81, positive predictive value (PPV) of 0.52, and negative predictive value (NPV) of 0.88; with an agreement rate of around 40% compared to histopathological assessment [28].

Mammography is a cornerstone in the diagnosis of breast cancer, thanks to its ability to detect microcalcifications; conversely, this feature is not helpful for evaluating the residual tumor burden. Kim et al. proved that the extent of the microcalcification poorly correlates with tumoral residua, because it could also characterize the NACT-induced necrosis [29]. Even if microcalcifications do not disappear post-NACT, MRI shows a better accuracy than MX [30].

Feliciano et al. emphasized that, if residual microcalcifications are found at the end of the treatment, it would be advisable to have surgery to remove them. In fact, though microcalcifications are not related to the persistence of tumoral cells for around 45% of cases, the absence of contrast enhancement in MRI imaging does not provide sufficient accuracy to avoid excision [31].

While in pre-treatment staging, mammography is recommended by the American College of Radiology (ACR) with a grade 9, as well as US and MRI; after-treatment, MX is downgraded to grade 8 and US to grade 7, contrary to MRI which remains at the highest grade [32].

In conclusion, there are multiple limitations to MX employment after NACT: MX does not adequately assess multicenter lesions; MX is not able to distinguish between lesions and changes in the tumor bed related to NACT, i.e., necrosis; microcalcifications are not useful for predicting the persistence of tumoral residua after therapy.

### 2.2. Ultrasound

Ultrasound has many advantages: it is a low-cost and non-invasive imaging modality that does not employ ionizing radiation. It allows the description of important tumor features such as the dimensions, morphology, and margins; and with some additional technologies, i.e., color-Doppler and elastography, it is also possible to evaluate tumor vascularization and stiffness [33]. For these reasons, the Chinese Anti-Cancer Association recommends US every two cycles of neoadjuvant treatment, in order to assess tumor response [34].

Concerning post-treatment evaluation, US is an effective technique, especially when the residual tumor is larger than 7 mm [35,36]. However, a reduction in vascular supply does not contribute to the assessment of the response [37]. In two studies, the US accuracy in predicting tumoral residual burden was 59.6% to 80%; conversely, for mammography it was 31.7% to 71% [38,39]. Keune et al. found that the correlation between the absence of lesions depicted on MX and US after treatment and pCR is of around 80% [26]. Furthermore, it is less accurate than MX for preoperatively detecting the size of ductal carcinoma in situ (DCIS) [40].

Elastography contributes to pretreatment staging with the assessment of tumor stiffness, which has been shown to be strongly correlated with tumor response; conversely, there is a lack of data for its usefulness post-treatment [41,42].

Likewise, we need further scientific evidence for the use of contrast-enhanced US (CEUS) in this field, although Cao et al. suggested that modifications in time–intensity curves could have a role in predicting tumoral response to NACT [43].

## 3. Role of MRI in Preoperative T Staging after NACT 

The refinement of magnetic resonance technique has led to its increased use in the field of breast cancer, due to its high sensitivity and high contrast resolution. MRI actually has many indications (i.e., the screening of women with a greater lifetime risk for breast cancer [44,45], discrepancy between clinical and imaging evaluation [46], study of breast silicone implants [47]) and, in the NACT setting, has the role of staging and monitoring the response to neoadjuvant treatment [46]. 

Compared to physical examination and other conventional imaging techniques (MX and US), MRI has a better performance in the assessment of tumor response in breast cancer patients undergoing NACT, because of its superiority in identifying tumor residua and in predicting pathologic complete response (pCR) [43,48,49,50,51,52,53,54]. Although the latter is not strictly necessary to perform a BCS, a better tumor response is linked to a better chance of success [55,56].

According to the European Society of Breast Cancer Specialists (EUSOMA), MRI should be performed in the preoperative evaluation of women who have received a new diagnosis of breast cancer oriented towards a BCS before the first course of NACT (but MRI must not significantly delay therapy initiation), for a greater anatomical definition of the index lesion and to assess the presence of any additional cancer foci [57].

The American College of Radiology (ACR) recommends MRI as being more sensitive and more specific compared to other methods, stressing the importance of a pre-treatment MRI to better estimate volumetric changes [32].

The best timing for post-NACT breast MRI, according to EUSOMA, should be two weeks after the last NACT cycle and within two weeks before surgery (treatment delay due to post-NACT MRI should not be longer than 1 month) [57].

### Evaluation of the Extent of Residual Disease

Various studies have demonstrated that MRI is the best method to assess residual malignancy, with around 90% sensitivity and from 60% to 100% of specificity, especially in multifocal and multicentric tumors, [43,48,49,50,51,52,53,54], even if sometimes it is likely to underestimate or overestimate the response [53,54,58,59,60,61].

Depending on the tumor subtype, MRI is more or less accurate: in fact, its accuracy is greater with invasive lobular carcinoma, HER2-positive, and TN tumors, and lower for the luminal A and B subtypes [62,63,64,65,66,67,68,69,70]. 

Pre-treatment non-mass enhancement and low nuclear grade are two other factors that affect MRI accuracy [71]. It should also be emphasized that MRI may overestimate the size of the residual tumor burden when there is an in situ component or when the response to treatment manifests as an area of fibrosis with scattered foci of contrast enhancement [72,73].

Additionally, the chemotherapeutic regimen can influence MRI features, making the evaluation more challenging; estrogen receptors (ER) modulators, antiangiogenic and taxane-containing agents may in fact lead to an underestimation [74,75].

Therefore, the ACRIN 6657 trial demonstrated that MRI was more accurate compared to other techniques (i.e., MX and physical examination), notably allowing measurement of the longest diameter of tumoral residua, which is closest to the final pathological size, and identifying pCR. Moreover, this trial concluded that the type of enhancement found at MRI, namely mass/non-mass and single or multiple, may have a great influence on the assessment; on the other side, little importance was given to the histology, presence of ductal carcinoma in situ (DCIS), and breast density at MX [76].

## 4. Evaluation of Lymph Node Involvement

The evaluation of node involvement is crucial for a correct assessment and must be carried out both at the time of diagnosis and after the conclusion of neoadjuvant treatment.

This valuation is important, even though imaging techniques do not allow the identification of “isolated tumor cells” (small aggregates of cells no larger than 0.2 mm or single cancer cells or a small clump of cells with less than 200 cells in one single histological section, pN0i+) or “micrometastases” (aggregate of contiguous tumor cells larger than 0.2 mm and/or more than 200 cells, but not larger than 2 mm, pNmi) [77,78], considering that these two conditions do not affect survival [79,80]. Although US is an operator-dependent method, at baseline, it is a very reliable imaging technique for the evaluation of the axilla, with a specificity of 88–98% and sensibility of 26–76% [81], due to its high resolution for evaluation of changes in the cortical zone of lymph nodes [82], and for the assessment of Berg levels I and II [32]; US also allows the realization of diagnostic insight into nodes with features of malignancy, guiding the execution of biopsies (fine needle aspiration or core biopsy). 

Meanwhile, MRI is necessary for a better characterization of Berg level III, internal mammary chains, supraclavicular lymph nodes, and for a comparison between the two axillary regions [32,83,84].

After neoadjuvant treatment, ultrasound was confirmed to be the best technique to evaluate the response and residual axillary disease, with around 70% sensitivity [85]. Alvarado and colleagues, in a study conducted on 150 women, observed that a normalized morphology post-NACT in previously node-positive disease is linked to better pathological response rates [86]. 

Shear wave elastography can be an additional evaluation tool to support ultrasound in the assessment of axillary status after NACT; in fact, a study conducted on 201 patients with pathologically-proven node-positive breast cancer suggested that the combination of the two imaging modalities can improve both the sensitivity and accuracy [87].

Some studies evaluated the accuracy of MRI in assessing lymph node status in patients treated with NACT; however, with discordant results, predominantly related to tumor histotype and molecular characteristics. Abel et al. conducted a retrospective study of a patient population with invasive lobular carcinoma (ILC) demonstrating that the accuracy of lymph node assessment was low compared to patients with invasive ductal carcinoma (IDC) [88]. In 2021, Samiei et al. performed a systematic review and metanalysis to compare the diagnostic performance of the different imaging techniques for the evaluation of axillary lymph node response after NACT in node-positive patients. Thirteen studies were included, representing a total of 2380 analyzed patients. Their conclusion was that ultrasound and MRI are limited for this assessment, identifying axillary residual disease in 77% and 78% and pCR in 50% and 58%, respectively [89].

For these reasons, if nodes were metastatic at baseline, it is recommended to perform a sentinel node biopsy or axillary node dissection [16,85,90].

## 5. Surgical Approach after NACT

### 5.1. Breast Surgery: Conserving Therapy or Not?

Surgery must be adapted to the response to neoadjuvant treatment and may consist of a total mastectomy, oncoplastic surgery, or breast-conservative surgery.

Over the years, the assessment of early-stage breast cancer has changed; initially, the first-line treatment was represented by mastectomy. For about 30 years, however, thanks to numerous randomized controlled trials and meta-analysis, [91,92,93,94,95,96], BCS has proven to be a reliable alternative, due to an equivalent long-term survival between the two treatments.

The aim of BCS is to achieve clear margins, to lower the risk of loco-regional disease, while achieving the best aesthetic outcome and preserving healthy tissue [1].

The risk of recurrence is high when the excision regards only the primary tumor. Various randomized controlled trials have demonstrated that radiation therapy affects the risk of local recurrence when associated with BCS: in 2005, the Early Breast Cancer Trialists’ Collaborative Group (EBCTCG) estimated that there was a 70% proportional reduction in loco-regional recurrences compared with BCS alone, with a 10-year risk of approximately 10%, and a minimal statistically significant reduction in mortality for women who received radiation therapy [97]. Moreover, an improvement in disease-free interval, as well as in overall survival, is obtained with the addition of adjuvant radiotherapy or hormonal therapy in the assessment of patients with early breast cancer.

Due to the large amount of data in the literature that supporting the safety of BCS over mastectomy in the treatment of breast cancer, women can choose between treatments, even following NACT [98,99,100]. 

**Figure 1:**
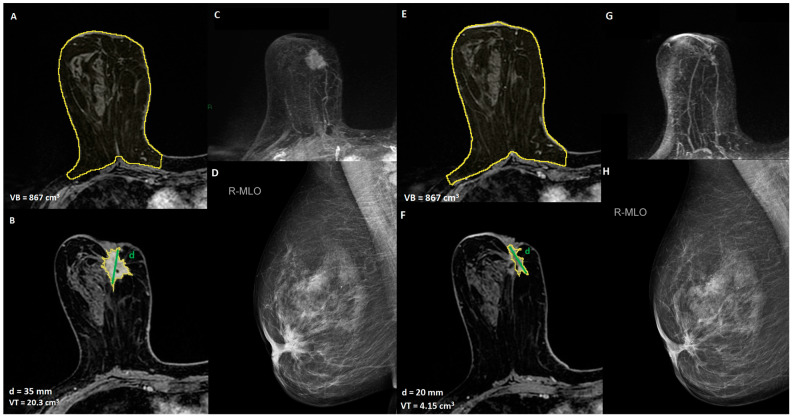
A 52-year-old women with unifocal HER2 positive invasive ductal carcinoma, infiltrating the nipple areola complex, with indication of breast-conservative surgery after NACT: (**A**,**B**) T1-weighted post-contrast MRI to define the extension of the disease and breast volume, VB = breast volume, VT = tumor volume, d = diameter of the lesion; (**C**) maximum intensity projection (MIP) pre-NACT image; (**D**) mediolateral oblique mammogram of the lesion, (**E**,**F**) T1-weighted post-contrast MRI to assess the response to NACT, (**G**) maximum intensity projection (MIP) post-NACT image, (**H**) mediolateral oblique mammogram of the lesion post-NACT.

Before choosing a breast-conserving treatment after neoadjuvant therapy, multiple clinical and histological criteria must be carefully evaluated, to ensure that the patient can be subjected to treatment without entailing an increased risk of loco-regional recurrence of disease; in particular, treatment should be proposed considering the size of the residual tumor, possible multifocality, the extension of suspicious microcalcifications associated with in situ carcinoma diagnosed on the biopsy, the volume ratio of the residual tumor to breast volume, and the localization of the tumor [98,99,101,102,103,104,105,106] (Figure 1). Straver et al., in a study conducted on 208 women, noticed that patients where better suited for BCS then mastectomy when MRI depicted a maximum size of the lesion not exceeding 30 mm on pretreatment, a dimensional reduction after treatment, and in HER2-positive and triple-negative subtypes [60]. 

**Figure 2:**
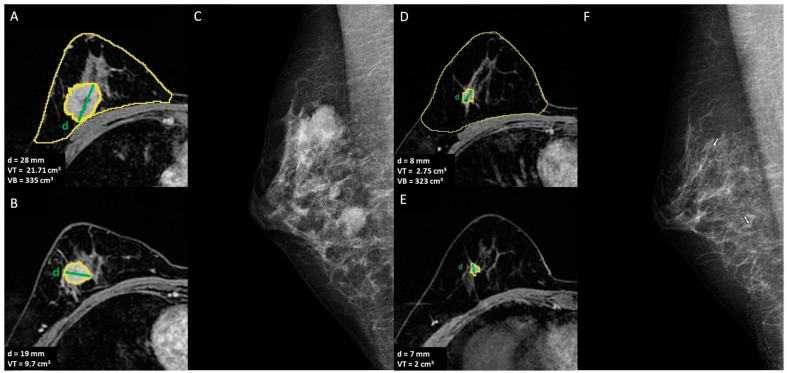
A 42-year-old women with multicentric invasive TN ductal carcinoma with indication of mastectomy after NACT: (**A**,**B**) T1-weighted post-contrast MRI to define the extension of the disease and breast volume, VB = breast volume, VT = tumor volume, d = diameter of the lesion; (**C**) mediolateral oblique mammogram shows the two lesions, (**D**,**E**) T1-weighted post-contrast MRI to assess the response to NACT, (**F**) mediolateral oblique mammogram of the two lesions post-NACT.

In order to optimize oncological and aesthetic outcomes in patients with large or multifocal tumors desiring breast conservation, oncoplastic surgery (OPS) is an option. The indication for OPS is a non-optimal response after NACT, for which a BCS with safe margins would either seem impossible or lead to major deformity [107]. Mastectomy remains indicated in patients with multicentric disease, widespread microcalcifications, or pathogenic variants of BRCA 1/2 genes [108,109,110] (Figure 2).

Although MRI is widely used to stage breast cancer in countries with a developed health care system, the current literature does not have a unitary view on the benefit in defining surgical planning brought by its routine preoperative use [111,112]. The fear is that MRI can overestimate the extent of the disease, leading to an increased number of unnecessary mastectomies [113]. In this regard, although NACT patients were excluded from the updated studies, it was demonstrated that preoperative MRI leads to a reduction in the rate of reoperation after conserving surgery, despite a slight increase in the rate of mastectomies [114,115].

If surgical margins are proven to be involved by the tumor in the pathological examination of resected specimens, it may be necessary for the patient to undergo re-excision surgery and, subsequently, radiotherapy. Mastectomy should be performed if clean margins cannot be obtained. A meta-analysis conducted in 2016 on eight trials, with a total of 3215 patients analyzed, stated that breast conserving surgery after NACT showed no significant difference in terms of the prevalence of local recurrence and five-year local recurrence-free survival rate when compared to mastectomy, thus allowing the possibility of performing a more conservative treatment without a loss in oncological outcomes [100]. Furthermore, conservative treatment is linked to greater aesthetic satisfaction of patients and less psycho-social morbidity [116,117,118]. 

Consequently, the choice between treatments must be made carefully on a case-by-case basis and must always aim to improve the patient’s outcome.

For these reasons, imaging, and in particular MRI, has a large role in the staging before and after neoadjuvant treatment, thus leading the surgical planning.

### 5.2. Axillary Surgery: Sentinel Lymph Node Biopsy, Lymphadenectomy, or Selective Axillary Dissection?

Axillary lymph node metastasis is an important prognostic factor in breast cancer and guides treatment planning.

At present, women with node-positive breast cancer often undergo NACT, which leads to elimination of lymph nodal disease in 40–70% of cases [119]. On the other hand, in women with clinically negative nodes pre-NACT, the use of the sentinel lymph node biopsy technique (SLNB) can accurately predict the axillary status in the post-treatment assessment [120,121,122]. The aim of SLNB is to overcome the standard surgical approach of axillary lymphadenectomy (AL) in node-positive breast cancer patients at diagnosis, preventing complications from the most radical surgical techniques [123,124,125]. 

**Figure 3:**
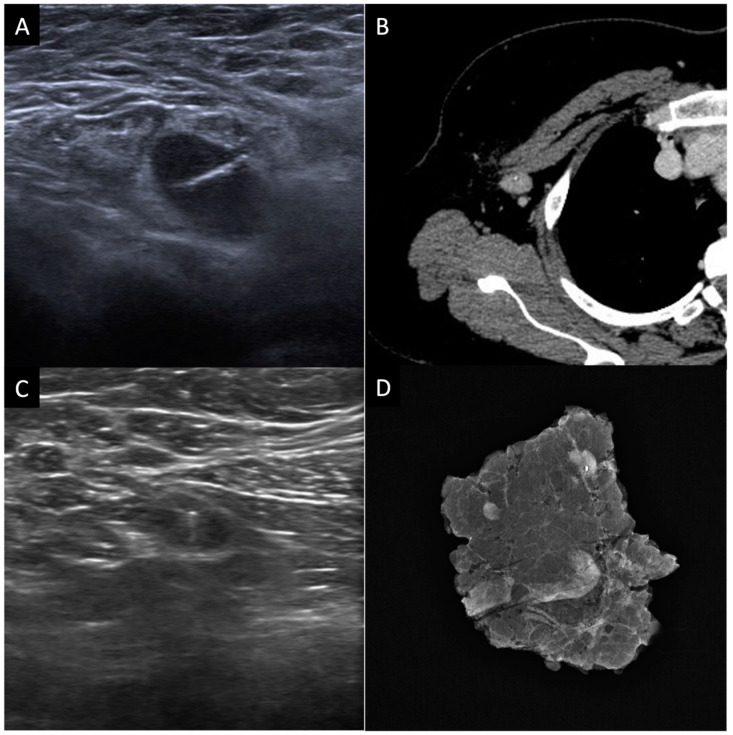
Selective axillary dissection technique (SAD): (**A**) ultrasound image of a clip marker placed in a histologically confirmed metastatic axillary lymph node before the beginning of NACT; (**B**) axial contrast enhanced CT image shows the clipped lymph node (CL); (**C**) ultrasound evaluation and identification of the CL before surgery; (**D**) surgical specimen radiograph to ensure that the CL has been removed along with the other lymph nodes.

Nevertheless, in this last cohort of patients, SLNB was proven to be not accurate in restaging axilla and, therefore, in selecting patients with complete lymph node disease regression after NACT, with a false negative rate (FNR) of 12.6–24.3 % [126,127]. To lower FNR, it is necessary to remove one or more lymph nodes in addition to sentinel lymph nodes; the greater the removal, the lesser the FNR [128]. The surgeon selects the lymph nodes to be removed by assessing their macroscopic and clinical characteristics during surgery, as well as their proximity to the sentinel lymph node. To better guide surgeon’s decision of which lymph node to remove beyond the sentinel nodes, several studies have proposed different modalities to help refine SLNB accuracy in the post-NACT setting: mandatory use of immunohistochemistry [127]; use of a dual mapping technique (both blue dye and radiolabeled colloid mapping agents) [16,126]; or the placement, before beginning of NACT, of a clip in the axillary node, which is proven to be metastatic at a core needle biopsy (CNB) [129,130,131,132]. In about 20% of patients, such a metastatic lymph node is not the same as the sentinel lymph nodes, because NACT can determine a modification of lymphatic drainage from breast neoplasm [129,131]. Therefore, if after NACT the clipped metastatic lymph node is not the same as the sentinel lymph nodes, it can be removed during surgery and histologically analyzed. The removal of the clipped lymph node (CL) was proved to reduce FNR of SLNB [131]; even in a subgroup analysis of the cohort of the ACOSOG Z1071 trial [129], patients with a metastatic clipped axillary node had less FNR than those without a clip. It has also been proposed that the SLNB associated with the removal of CL, the so called “selective axillary dissection” (SAD) (Figure 3), can therefore enable the selection of the lymph nodes, to allow analysis in a more correct and repeatable way, increasing the accuracy in the evaluation of lymph nodes pathological response to NACT; this allows the selection of patients who can safely avoid AL, with a significant positive effect on their quality of life [133].

### 5.3. Surgery Omission after NACT

Due to the constant progress in neoadjuvant therapies and imaging techniques, the possibility of omitting surgery in breast cancers is now being considered as a viable alternative.

Approximately 19% of patients treated with neoadjuvant chemotherapy achieve a pCR, even if this probability varies between the different subtypes: for hormone-positive tumors it is 8.3%, for HER2+/hormone-positive tumors it is 18.7%, for TN it is 31.1% and for HER2+/hormone negative it is 38.9% [134].

In patients with an excellent response to neoadjuvant chemotherapy, it has long been proposed to avoid surgery in favor of a radiation therapy alone, with disappointing results in terms of loco-regional recurrence (21–47%) [135,136,137,138,139]. However, these studies had suboptimal methodologies: selection of patients based on clinical response alone, lack of selection based on tumor subtypes, and no use of a radiological guidance biopsy to document the pathological response. In particular, a major obstacle to the possibility of omitting surgery is related to the suboptimal specificity of the imaging methods in predicting sufficiently accurately the absence of residual disease after neoadjuvant chemotherapy, and therefore today surgery remains indispensable for verifying the response to the NACT in the surgical specimen [53,139,140,141].

Multiple clinical feasibility trials were performed or are currently ongoing all around the world to solve this problem: their goal is to identify key elements to guarantee the accuracy and safety for patients selected for non-operative management [142,143,144,145,146,147,148]. These include the possibility of sampling residual abnormalities thanks to image-guided percutaneous biopsy, which helps to evaluate tumoral response after-treatment [149]. The main question remains which is the best technique or combination of techniques to combine with vacuum-assisted core biopsy (VACB), to reduce false-negative rates and to augment negative predictive values. If these studies lead to the expected results, they will probably induce a drastic change in the way we manage breast cancer after NACT, both from a therapeutic and diagnostic point of view.

## 6. New Perspectives: Ultrafast Breast MRI, Contrast-Enhancement Mammography, Radiomics, and Machine Learning

Ultrafast MRI is an emerging technique that is increasingly used in clinical practice, as the first studies carried out on its non-inferiority are very promising. Its goal is to reveal the early wash-in of contrast material at high temporal resolution, usually less than 6–7 s. Unlike conventional kinetic curves, these new sequences allow obtaining early wash-in kinetic curves through rapid sequential imaging taken in the first 120 s after contrast injection [150].

A limit to the widespread use of ultrafast MRI is the need for specific coils and sequences that enable a high temporal resolution in parallel to a diagnostic spatial resolution. The strength of this technique is its ability to detect the early contrast enhancement that characterizes breast cancers [151].

A prospective study, published in 2022 and conducted on 50 patients that underwent neoadjuvant therapy, demonstrated that the wash-in slope at initial ultrafast DCE-MRI can be used as a predictive factor of pCR, reporting a sensitivity and specificity of 94% and 59%, respectively (WIS cut-off value equal to 1.6% per second) [152].

Moreover, a recently published Korean study investigated the association between kinetic features obtained from ultrafast MRI and pCR in 256 women with invasive breast cancer undergoing NACT and surgery, discovering an independent association between a higher volume ratio between two different time points of lesion enhancement and pCR in TN tumors [153].

Though there is still little data in the current literature, some recent studies confirmed that the diagnostic performance of contrast-enhanced mammography (CEM) in the valuation of pathological response after neoadjuvant therapies is very encouraging. 

Moustafa and colleagues, in 2019, also evaluated the use of a quantitative mathematical objective tool for this purpose, in comparison to RECIST 1.1 criteria and a subjective visual analysis, on 42 women: this tool was effective for assessing dimensional changes, but also for obtaining information about the constitutional differences of tumor residua after NACT and to eliminate bias in the evaluation [154].

In a prospective study conducted in 2017, the reported specificity, sensitivity, NPV, and PPV in the prediction of the response to therapy depicted by CEM were 91%, 40%, 80%, and 62.5%, respectively, with a sensitivity and specificity for complete response of 100% and 83% [155].

Promising results were also obtained when CEM was compared with MRI; in fact, a meta-analysis conducted on 24 studies in 2020 concluded that the two imaging techniques have an equal specificity, whilst CEM has a better sensitivity than MRI [156].

Radiomics represents the future of imaging, and the focus of the scientific community on these new technologies is increasing, including on breast cancer, i.e., with several studies whose purpose is to explore their applications in differential diagnosis or prognosis [157,158,159].

Some interesting studies have also been performed to predict the tumor response after neoadjuvant treatment through imaging omics, with surprising results. For this purpose, Zhuang et al. established a nomogram able to guide therapeutic decisions, thanks to an analysis based on multiparametric MRI radiomics combined with clinico-pathological factors [160].

A multicenter study published in 2019 showed that a radiomic signature based on multiparametric MRI and combinations of sequences achieved a higher AUC (0.79) compared with a single-sequence model. It also showed good results in hormone receptor positive, HER2-negative, and triple negative tumors [161].

Cain et al. studied a multivariate model based on machine learning and able to obtain features to predict pCR after NACT on pre-treatment DCE-MRI, in patients with HER2 over-expressing and triple negative cancers [162].

Although MRI parameters have been the most studied, other techniques also show encouraging results, i.e., as demonstrated by a study by Antunovic et al. on positron emission tomography (PET)/CT-based models [163].

## 7. Conclusions

The ability of imaging to predict tumoral response and to assess residual burden is crucial in delivering tailored treatments to women with breast cancer undergoing neoadjuvant therapies. This entails the reduction of unnecessary treatments and associated toxicity, and the orientation towards conservative surgical treatment, to achieve the best oncological outcome. Knowledge is improving so quickly that important information on the biological characteristics of tumors can already be obtained, thanks to the current advanced imaging technologies, of which MRI is the most representative. Further new technologies, while being at the initial stages, are providing encouraging results in this field and will surely change the assessment of these patients.
